# The Long Non-Coding RNA Gene *AC027288.3* Plays a Role in Human Endometrial Stromal Fibroblast Decidualization

**DOI:** 10.3390/cells13090778

**Published:** 2024-05-02

**Authors:** Rupak Thapa, Kevin Marmo, Liang Ma, Donald S. Torry, Brent M. Bany

**Affiliations:** 1 Department of Physiology, Southern Illinois University School of Medicine, Carbondale, IL 62901, USA; rupak.thapa@siu.edu (R.T.);; 2 Division of Dermatology, Department of Medicine, Washington University School of Medicine, St. Louis, MO 63110, USA; 3 Department of Medical Microbiology, Immunology and Cell Biology, Southern Illinois University School of Medicine, Springfield, IL 62702, USA

**Keywords:** decidualization, endometrium, lncRNA

## Abstract

During the secretory phase of the menstrual cycle, endometrial fibroblast cells begin to change into large epithelial-like cells called decidual cells in a process called decidualization. This differentiation continues more broadly in the endometrium and forms the decidual tissue during early pregnancy. The cells undergoing decidualization as well as the resulting decidual cells, support successful implantation and placentation during early pregnancy. This study was carried out to identify new potentially important long non-coding RNA (lncRNA) genes that may play a role in human endometrial stromal fibroblast cells (hESF) undergoing decidualization in vitro, and several were found. The expression of nine was further characterized. One of these, *AC027288.3*, showed a dramatic increase in the expression of hESF cells undergoing decidualization. When *AC027288.3* expression was targeted, the ability of the cells to undergo decidualization as determined by the expression of decidualization marker protein-coding genes was significantly altered. The most affected markers of decidualization whose expression was significantly reduced were *FOXO1*, *FZD4*, and *INHBA*. Therefore, *AC027288.3* may be a major upstream regulator of the WNT-FOXO1 pathway and activin-SMAD3 pathways previously shown as critical for hESF decidualization. Finally, we explored possible regulators of *AC027288.3* expression during human ESF decidualization. Expression was regulated by cAMP and progesterone. Our results suggest that *AC027288.3* plays a role in hESF decidualization and identifies several other lncRNA genes that may also play a role.

## 1. Introduction

The transdifferentiation or decidualization of endometrial fibroblasts into epitheloid-like decidual cells is critical in establishing pregnancy in many species. It begins during the secretory phase of the uterine cycle in humans and continues during early pregnancy, ultimately forming the decidual tissue [1]. The decidual tissue supports the development of the conceptus during early pregnancy, including the development of the placenta, and thus is a primary driver of pregnancy health success [2]. Initially, perivascular fibroblast cells begin to undergo decidualization as progesterone levels rise during the secretory phase of the menstrual cycle. This transdifferentiation then spreads more broadly in the endometrium during early pregnancy. Human endometrial stromal fibroblast (hESF) decidualization is often studied in vitro using cells isolated from the endometrium of non-pregnant or term placenta of pregnant patients [3].

Long non-coding RNAs (lncRNAs) commonly refer to RNA transcripts that are greater than 200 nucleotides that do not encode proteins that are produced by RNA pol I, II, or III [4,5]. However, it has more recently been suggested that it would be more useful if lncRNAs were defined as at least 500 nt long and generated mainly by RNA pol II [4]. Like protein-coding genes, most lncRNA genes have introns and exons and can undergo similar RNA maturation steps. The current release of the Human Genome (Gencode release 45, [6]) includes 20,424 LncRNA and 19,395 protein-coding genes. The Ensembl Database [7] classifies lncRNAs into different types with respect to their location and orientation relative to protein-coding genes, including 3′ overlapping ncRNA, antisense, macro lncRNA, non-coding, retained intron, sense intronic, sense overlapping, and long intergenic ncRNA (lincRNA). Like protein-coding genes, Pol II-transcribed lncRNAs are spliced, bearing polyadenylated tails at the 3′-end and 7-methyl guanosine caps at the 5′-end. In contrast, lncRNAs transcribed by other means lack caps and poly-A tails. LncRNAs are believed to be a functionally essential part of the mammalian transcriptome, and their functions are diverse. For example, lncRNAs are known to play roles in many biological processes, such as chromatin modification, transcriptional or translational regulation, scaffolds for proteins, and microRNA “sponges” [8,9,10,11]. However, the function of most lncRNAs remains to be defined.

Previous findings have demonstrated the potential functions of some select lncRNAs in the endometrium under pathological and physiological conditions. Abnormal expression of some lncRNAs in the endometrium or decidua is associated with fertility or gestational abnormalities such as endometriosis [12,13,14,15], recurrent implantation failure [16], and preeclampsia [17]. Some examples of the potential physiological role of lncRNA’s in hESF decidualization so far include *HAND2-AS1* [18], *MALAT1* [15], *ENSG00000230699* (LncSAMD11-1:1, [19], *HK2P1* [20], *TUNAR* [21], *LUCAT1* [22], and *LINC00473* [23]. Many lncRNAs show differential expression in the mouse endometrium during decidualization [24,25]. However, lncRNAs shown to play a functional role in mouse endometrial fibroblast decidualization are few, including *Gm7932* [24], *Hand2-os1* [25], and *Dio3os* [26]. This study aimed to determine common lncRNA genes differentially expressed during hESF decidualization in vitro from RNA-seq data from two different models commonly used to study in vitro decidualization. We identified a large number of differentially expressed lncRNA genes during hESF decidualization in vitro and further characterized the expression of nine. Of these, the function of *AC027288.3* was assessed and was determined to play a role in hESF decidualization in vitro.

## 2. Methods and Materials

### 2.1. Cell Isolation and Culture

Human ESF cells were isolated from the term placentae of normal pregnant patients (with the approval of the Institutional Review Board Committee of Southern Illinois University School of Medicine) and cultured using methods described previously [27]. Some hESF cells used in this study were a generous gift from Asgi Fazleabas (Michigan State University). Briefly, cells were cultured in 10% heat-inactivated charcoal-stripped fetal bovine serum (hicsFBS) (Neuromics, Edina, MN, USA), and once the cells reached ~70% confluence, they were incubated for 24h with media containing 2% hicsFBS. The cells were treated with a cocktail (estradiol, medroxyprogesterone, and cAMP; EPC) in media containing 2% hicsFBS to induce decidualization as described elsewhere [27]. Other treatments included human recombinant BMP2, estradiol, medroxyprogesterone acetate, and cAMP analog, as described elsewhere [27].

### 2.2. Poly A+ RNA-Seq Library Preparation and Sequencing

Human ESF cells isolated from 3 patients (N = 3) were treated with vehicle (VEH; 0.1% ethanol) and EPC for 48 h. Total RNA was isolated using a Direct-zol™ RNA MiniPrep Plus Kit (Zymo Research, Irvine, CA, USA) as directed by the manufacturer using TRIzol™ (ThermoFisher Scientific, Waltham, MA, USA). The RNA samples were sent to Cofactor Genomics (St. Louis, MO, USA) for RNA-seq library construction and sequencing. Total RNA sample quantity and quality were assessed as directed by the manufacturers using Qubit RNA Assay (ThermoFisher Scientific) and Agilent Bioanalyzer RNA (Agilent Technologies, Santa Clara, CA, USA) kits, respectively. Libraries were constructed using a KAPA Stranded mRNA-Seq Kit as suggested by the manufacturer (KAPA Biosystems, Wilmington, MA, USA). Libraries were sequenced as single-end 75 base-pair reads on an Illumina NextSeq500 following the manufacturer’s protocols (Illumina, San Diego, CA, USA). The sequencing files have been submitted to NCBI (Project PRJNA1068120).

### 2.3. RNA-Seq Analysis

The Poly A+ RNA-seq reads were mapped to the Genome Reference Consortium Human Build 38 (GRCh38.p14, Release 45) downloaded from Gencode [6], using Spliced Transcripts Alignment to a Reference (STAR) [28]. Transcript read counts were then determined using the software package HTSeq (version 2.0.5) [29]. Differential gene expression between vehicle- and EPC-treated cells isolated from 3 patients (N = 3) was determined using the Bioconductor package edgeR [30] as patient-matched samples as recommended in the user’s guide [31]. The false discovery rate (FDR) of differentially expressed genes (DEGs) was estimated using the Benjamini and Hochberg option, and those with an absolute fold-change (|FC|) ≥ 2 and an FDR < 0.01 were considered to be significantly differentially expressed between the vehicle and EPC-treated groups. To compare our Poly A + RNA-seq data from hESF cells isolated from pregnant patients to those isolated from non-pregnant patients, we also reanalyzed the raw data from previous studies [32,33] (PRJNA368634) in a similar fashion. The data included hESF cells from non-pregnant patients with non-targeting, PGR-targeting, or FOXO1-targeting siRNA prior to treatment with vehicle or EPC for 72 h. Where possible, all RNA-seq data were annotated with HNGC gene symbols from an Ensembl GeneID using Ensembl BioMart [7]. When this was impossible, the Human ENSEMBL Gene ID converter available at www.biotools.fr (accessed 10 January 2024) was used. Venn diagrams and Volcano plots of -Log_10_(FDR) versus Log_2_(FC) were prepared using GraphBio [34]. Bigwig files were produced using the BamCoverage function of Deeptools2 [35] using bins per million normalization (bin size = 20) and displayed on the UCSC Genome Browser [36].

### 2.4. Reverse Transcription-Quantitative PCR (RT-qPCR)

Total RNA isolation, cDNA preparation, primer validation, and real-time qPCR were carried out as previously described [27]. The qPCR data were analyzed using the ΔΔCt method (31) using *RPLP0* (36B4) as the housekeeping gene as previously used for human endometrial stromal cell decidualization experiments [27,37]. The validated primer sequences are in Table 1.

### 2.5. Cell Fractionation

After trypsinization, the cells were centrifuged at 100× *g* for 5 min at 4 °C to obtain a cell pellet. The cells were washed and re-centrifuged thrice with Dulbecco’s PBS (D-PBS). The final pellets of cells were gently resuspended in 0.1 mL hypotonic solution (10 mM Tris-HCl, 15 mM KCl, 1 mM EDTA, 0.5 mM dithiothreitol, pH 7.4) and incubated on ice for 3 min. Then, 100µL of lysis buffer (hypotonic solution containing 0.1% NP-40) was added to the cell suspension, and the mixture was gently mixed and placed on ice for 4 min. The entire lysate was overlaid on top of 0.5 mL of 24% sucrose solution (10 mM Tris-HCl, 15 mM KCl, 24% sucrose, 0.15 mM spermine, 0.5 M spermidine, 0.5 mM dithiothreitol, pH 7.5) and subjected to centrifugation at 6000× *g* for 10 min at 4 °C. The resulting upper 0.3 mL containing the cytoplasmic fraction was carefully transferred to a new tube, and the nuclear pellets were retained. The nuclear pellets were washed with D- PBS containing 0.1 mM EDTA, followed by centrifugation at 1000× *g* for 10 min at 4 °C. The nuclear pellet was resuspended in 0.3 mL of nuclear lysis buffer (20 mM HEPES, 0.5 M NaCl, 0.5 mM dithiothreitol and incubated on ice for 30 min. The nuclear and cytoplasmic fractions were used to prepare total RNA as above or subjected to Western blot analysis.

### 2.6. Protein Extraction and Western Blot Analysis

Equal volumes of cytoplasmic and nuclear fractions were subjected to reducing 12% SDS-PAGE and then transferred onto Immobilon-FL membranes (Millipore; Billerica, MA, USA) using methods we described elsewhere [38]. Western blot analysis was carried out using mouse anti-GAPDH (Sigma-Aldrich Cat# G8795, RRID: AB_1078991, St. Louis, MO, USA) and mouse anti-SNRNP70 IgG (Santa Cruz Biotechnology Cat# sc-390899, RRID: AB_2801569, Santa Cruz, CA, USA) primary antibodies followed by Alexa Fluor 790 goat anti-Mouse IgM (Jackson ImmunoResearch Labs Cat# 115-655-075, RRID: AB_2338943, West Grove, PA, USA) and Alexa Fluor 680 Anti-mouse IgG (Jackson ImmunoResearch Labs Cat# 115-625-205, RRID: AB_2338938) secondary antibodies, respectively. Briefly, after immersion of the membranes in Li-Cor Intercept blocking buffer (Li-Cor) for 2 h, the membranes were incubated with non-immune goat IgG or primary antibodies in 0.5× blocking buffer diluted in PBS containing 0.1% Tween-20 (PBST) for one hour with gentle shaking. After washing three times for 10 min with PBS containing 0.1% Tween-20, the membranes were incubated for one hour with secondary antibody in PBS containing 5% BSA (Cell Signaling, Cat# 9998, Danvers, MA, USA), 0.1% Tween 20, and 0.001% SDS. The membranes were washed three times in PBST and finally with PBS. Finally, membranes were scanned using a Li-Cor Odyssey Infrared scanner (Li-Cor, Lincoln, NE, USA).

### 2.7. ChIP-Seq Data Reanalysis

Previously published FOXO1 ChIP-seq data (NCBI: PRJNA285913) [32] were uploaded to the Galaxy web platform at usegalaxy.org to analyze the data [39]. After removing duplicate reads and the remaining reads passed a quality filter, the clean reads were mapped to the human genome (GRCh38/hg38) using Bowtie2 [40] with default parameters. Peak calling was performed using MACS2 callpeak [41] with default parameters. MACS2 bdgcmp was used to subtract the input control background from the FOXO1 ChIP data. After conversion to BigWig files, the data were displayed on the UCSC Genome Browser [36]. We also downloaded a BED file from the peak-browser webpage (cell type class; uterus; cell type: endometrial stromal cells) of Chip Atlas [42,43]. The following ChIP-seq NCBI accession numbers were visualized on the Integrative Genomics Viewer (IGV) application [44]: SRX1048946 (FOSL2), SRX1048948 (FOXO1), SRX35033584 (GATA2), SRX5088243 (NCOA2), SRX372174 (NR2F2), SRX1048945 (PGR), and SRX1435941 (ZBTB16) which contain ChIP-seq data on hESF cells undergoing EPC-induced decidualization in vitro [32,33,45,46,47,48].

### 2.8. FOXO1 ChIP-PCR

Chromatin preparation, immunoprecipitation, RT-qPCR, and percent input calculations were carried out using methods previously described in detail [49] using control rabbit IgG (Cell Signaling Technology Cat# 2729, RRID:AB_1031062) and rabbit anti-human FOXO1 (Cell Signaling Technology Cat# 2880, RRID:AB_2106495). For ChIP qPCR primer validation, optimal annealing temperatures and PCR efficiencies were determined, and melt-curve analyses after qPCR verified the presence of single amplicons without primer dimers using diluted input samples. The sequence of the ChIP qPCR primers is in Table 2.

### 2.9. Lentivirus Preparation and AC027288.3 Knockdown

VSVG pseudotyped lentiviral particles containing shRNA targetting AC027288.3 were prepared, concentrated, titered, and used as previously described [50]. The AC027288.3 shRNA target sequence used in this study was GCATCTACAATCTGTATTATT, while the non-targeting sequence was CCTAAGGTTAAGTCGCCCTCG. For experiments, cells were plated, transduced with lentivirus, and treated exactly as previously described [27].

### 2.10. Statistical analysis

Graphs and statistical analysis of the RT-qPCR results for changes in RNA expression were prepared using Sigmaplot software version 15.X (SPSS Inc., Chicago, IL, USA) as previously described [27]. For ChIP-PCR results, paired *t*-tests were used. Statistical significance in the results was noted at the *p* < 0.05 (a), *p* < 0.01 (b), *p* < 0.005 (c), or *p* < 0.001 (d) levels.

## 3. Results

### 3.1. RNA-Seq Analysis

**Figure 1:**
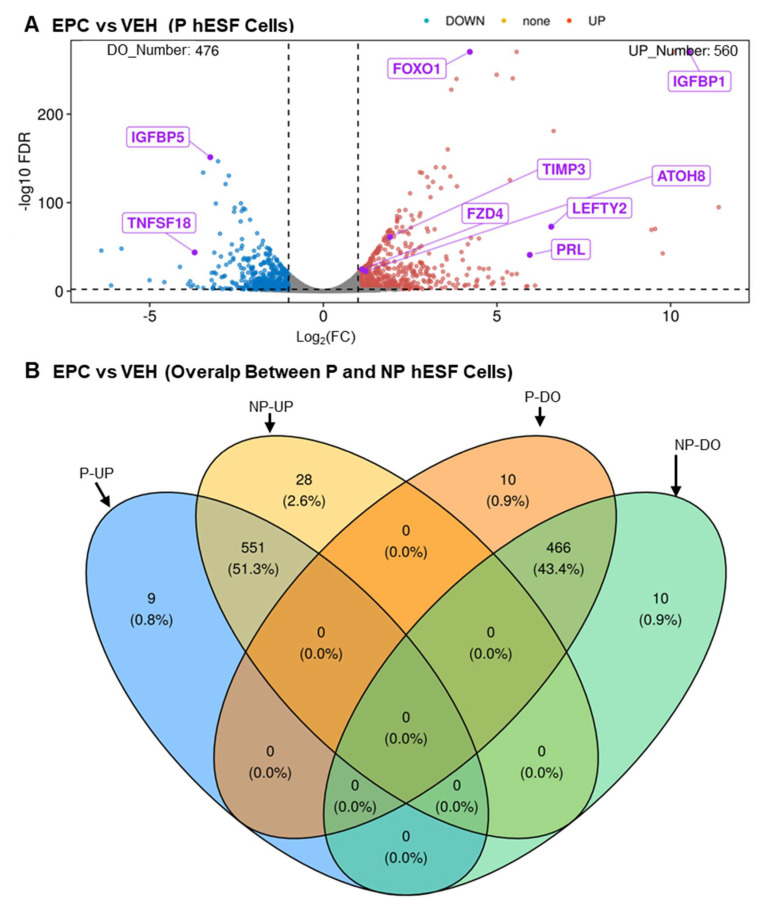
RNA-seq results reveal a large number of protein-coding genes are differentially expressed during hESF cell decidualization. (**A**) Volcano plot of RNA-seq results of hESF cells isolated from pregnant patients treated with vehicle (not undergoing decidualization) or EPC (undergoing decidualization). Note that fold-change represents EPC- compared to VEH-treated cells. (**B**) Venn diagram comparing the RNA-seq results of hESF cells isolated from pregnant (P) patients to those isolated from non-pregnant (NP) patients. Abbrev: FDR, false-discovery rate; FC, fold change EPC- compared to VEH-treated cells; UP increases during decidualization; DO, decreases during decidualization. Data are provided in Supplemental Tables S1–S3.

In order to find the identity of new lncRNA genes that may play a role in human endometrial decidualization, we carried out RNA-seq analysis of the transcriptome changes between EPC- (undergoing decidualization) and vehicle-treated (not undergoing decidualization) hESF cells isolated from pregnant patients. We then compared it with our reanalysis of previously published data obtained from hESF cells from non-pregnant patients. The complete listing of data obtained for protein-coding genes for hESF cells from pregnant and non-pregnant patients can be found in Supplemental Tables S1 and S2, respectively. A volcano plot of RNA-seq analysis of the differential mRNA expression between EPC- and vehicle-treated hESF cells from pregnant patients is shown in Figure 1A. The number of protein-coding genes that were significantly upregulated and downregulated during the decidualization of the pregnant hESF cells was 560 and 476, respectively. A comparison between RNA-seq analysis results between hESF cells from pregnant and non-pregnant patients is shown in the Venn diagram in Figure 1B. The number of protein-coding genes significantly upregulated and downregulated during decidualization in common between pregnant and non-pregnant hESF cells during decidualization was 551 and 466, respectively. The complete listing of the protein-coding genes in the different categories of the Venn diagram is in Supplemental Table S3. The results show that many of the previously published protein-coding genes known to be differentially expressed during hESF decidualization were shared between the two RNA-seq datasets. For example, these genes include those whose expression increases (e.g., *FOXO1*, *IGFBP1*, *PRL*, *TIMP3* [32], *ATOH8*, *LEFTY2*, and *FZD4* [27]) and decreases (e.g., *IGFBP5* [51]) during hESF decidualization.

**Figure 2:**
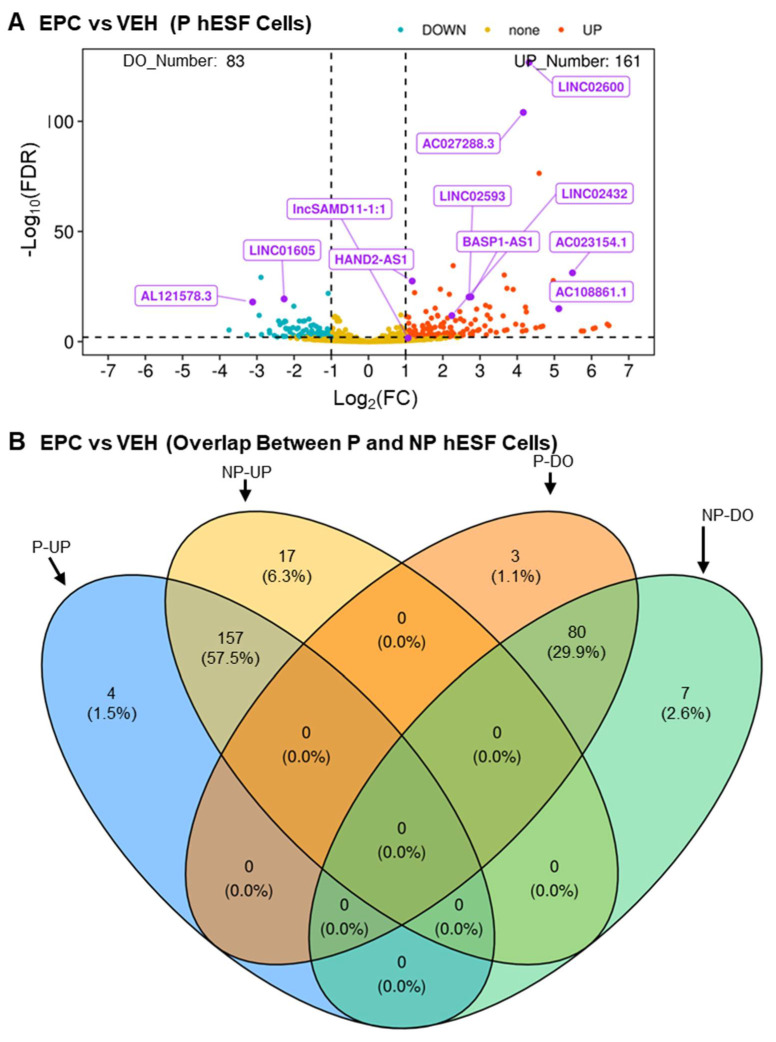
RNA-seq results reveal a moderate number of long non-coding genes are differentially expressed during hESF cell decidualization. (**A**) Volcano plot of RNA-seq results of hESF cells isolated from pregnant patients treated with vehicle (not undergoing decidualization) or EPC (undergoing decidualization). (**B**). Venn diagram comparing the RNA-seq results of hESF cells isolated from pregnant patients (P) to those isolated from non-pregnant (NP) patients. Abbrev: FDR, false-discovery rate; FC, fold change EPC- compared to VEH-treated cells; NC, no change; UP increases during decidualization; DO, decreases during decidualization. Data are provided in Supplemental Tables S4–S6.

The complete listing of data obtained for LncRNA genes for hESF cells from pregnant and non-pregnant patients can be found in Supplemental Tables S4 and S5, respectively. A volcano plot of RNA-seq analysis of the differential lncRNA expression between EPC- (undergoing decidualization) and vehicle-treated (not undergoing decidualization) hESF cells from pregnant patients is shown in Figure 2A. The number of lncRNA genes that were significantly upregulated and downregulated during decidualization was 161 and 83, respectively. A comparison between RNA-seq analysis results between hESF cells from pregnant and non-pregnant patients is shown in the Venn diagram in Figure 2B. The number of lncRNA genes significantly upregulated and downregulated in common between pregnant and non-pregnant hESF cells during decidualization was 157 and 80, respectively. The complete list of the LncRNA genes in the Venn diagram is in Supplemental Table S6. The results show that a couple of the previously published lncRNA genes known to be differentially expressed during hESF decidualization as common upregulated lncRNA genes, including *HAND2-AS1* [18] and *LNCSAMD11-1:1* (ENSG00000230699, ENST00000448179) [19].

### 3.2. Verifying Differential Expression of 9 Novel LncRNA during hESF Decidualization

**Figure 3:**
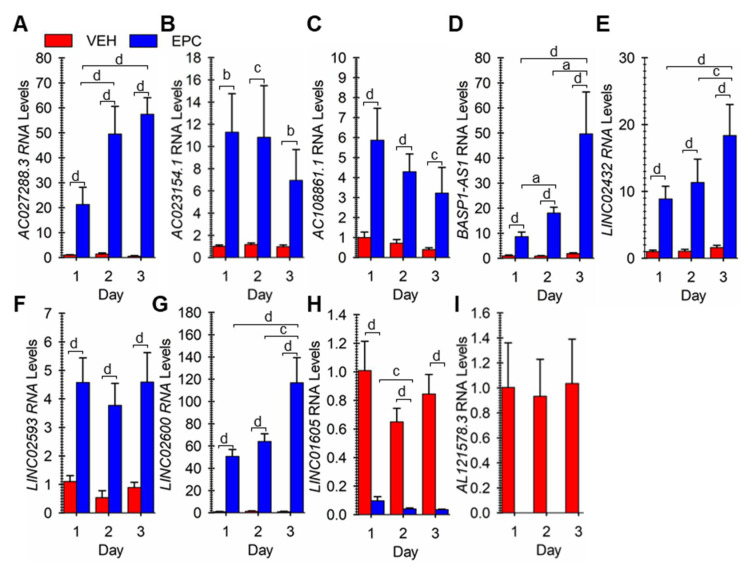
Verification of differential expression of nine lncRNA genes during hESF decidualization. Cells were treated vehicle (VEH) or stimulated to undergo decidualization with EPC for 1, 2, or 3 days, and relative RNA levels for (**A**) *AC027288.3*, (**B**) *AC023154.1*, (**C**) *AC108861.1*, (**D**) *BASP1-AS1*, (**E**) *LINC02432*, (**F**) *LINC02593*, (**G**) *LINC02600*, (**H**) *LINC01605*, and (**I**) *AL121578.3* were determined by reverse-transcription quantitative polymerase chain reaction. Bars represent mean ± SEM using cells from 6 independent patients (N = 6). Statistical significance denoted as a, b, c, and d represent *p* < 0.05, 0.01, 0.005, and 0.001, respectively.

From the results, we focused further on nine different random lncRNAs that were similarly differentially expressed in both pregnant and non-pregnant hESF cells undergoing decidualization. For all 9, the Ensembl GeneID and all possible TranscriptIDs and the primary transcript expressed as determined by looking at the RNA-seq data on the UCSC genome browser (Supplemental Figures S1–S9) are summarized in Supplemental Table S7. To verify the differential expression of these lncRNAs, we carried out RT-qPCR on cells incubated with vehicle or EPC for 1, 2, or 3 days. For lncRNAs *AC027288.3*, *AC023154.1*, *AC108861.1*, *BASP1-AS1*, *LINC02432*, *LINC02593,* and *LINC02600* expression was significantly greater in EPC-treated cells compared to vehicle-treated cells on each day examined (Figure 3A–G). The expression of LINC01605 was significantly greater in vehicle-treated cells compared to EPC-treated cells on each day examined (Figure 3H). Finally, *AL121578.3* RNA expression was only detected in vehicle-treated cells at 1–3 days (Figure 3I).

### 3.3. Localization of LncRNA Expression

**Figure 4:**
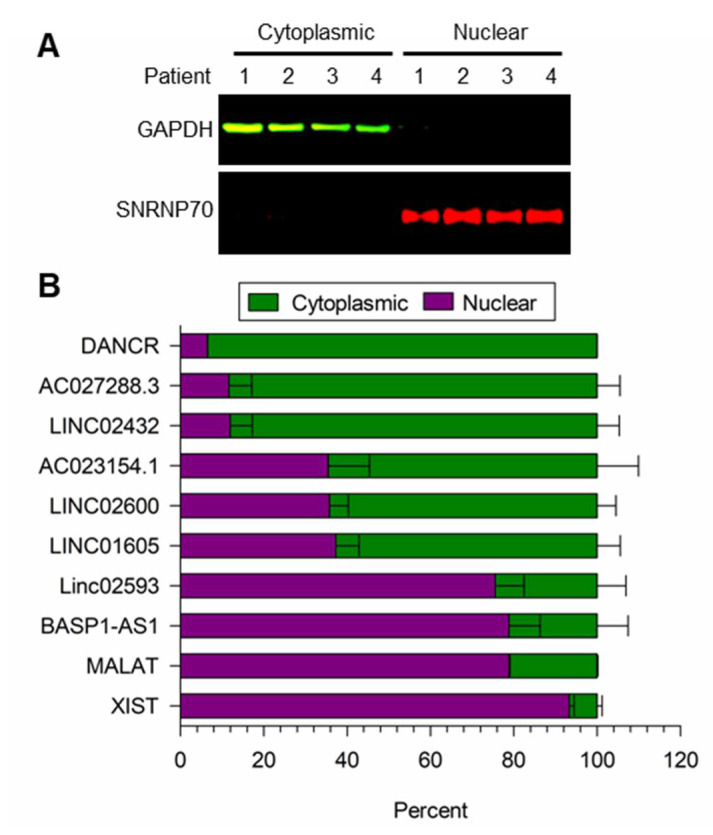
Cellular localization of lncRNA expression in EPC-treated hESF cells. (**A**) Western blot analysis of cytoplasmic and nuclear fractions for cytoplasmic GAPDH and nuclear SNRNP70 proteins. (**B**) RNA levels in cytoplasmic versus nuclear fractions for *DANCR* (mainly cytoplasmic), *MALAT* plus *XIST* (nuclear), along with *AC027288.3*, *LINC02432*, *AC023154.1*, *LINC02600*, *LINC01605*, *LINC02593*, and *BASP1-AS1* lncRNAs. Bars represent mean ± SEM using cells from 4 independent patients (N = 4).

We validated the fractionation method to isolate nuclear and cytoplasmic lncRNA in cells treated with EPC for 48 h. As shown in Figure 4A, the cytoplasmic GAPDH and nuclear SNRNP70 protein markers were only detected in the nuclear and cytoplasmic fractions. As shown in Figure 4B, the known cytoplasmic *DANCR* and nuclear *MALAT* plus *XIST* lncRNA markers [52,53,54] were predominantly detected in the cytoplasmic and nuclear fractions, respectively. Additionally, shown in Figure 4B, *AC027288*.3 and *LINC02432* were predominantly cytoplasmic, while *LINC02593* and *BASP1*-*AS1* were mainly nuclear. Finally, significant amounts of *AC023154*.*1, LINC02600*, and *LINC01605* were seen in both the nuclear and cytoplasmic fractions.

### 3.4. Steroid and cAMP Control of LncRNA Expression

**Figure 5:**
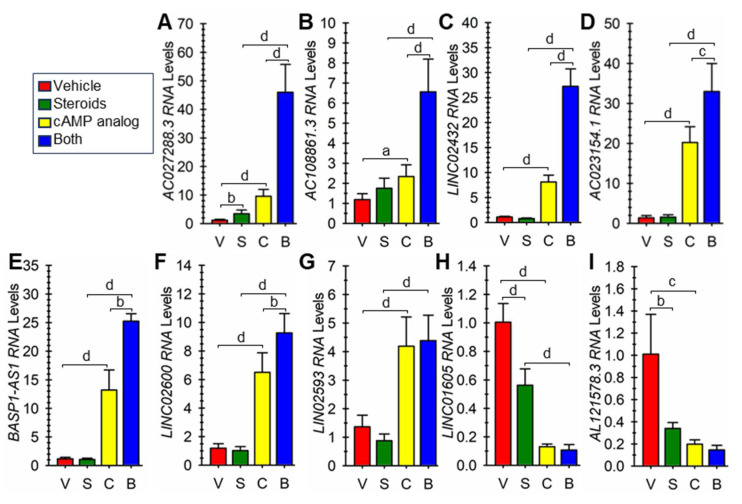
Effects of steroids versus cAMP components of EPC on lncRNA expression. Cells were incubated with vehicle (V), steroids (S), cAMP analog (C), or both steroids plus cAMP analog (B) for 48 h. Relative lncRNA levels for (**A**) *AC027288.3*, (**B**) *AC023154.1*, (**C**) *AC108861.1*, (**D**) *AL121578.3*, (**E**) *BASP1-AS1*, (**F**) *LINC01605*, (**G**) *LINC02432*, (**H**) *LINC02593*, and (**I**) *LINC02600* were determined by reverse-transcription quantitative polymerase chain reaction. Bars represent mean ± SEM using cells from 6 independent patients (N = 6). Statistical significance denoted as a, b, c, and d represent P less than 0.05, 0.01, 0.005, and 0.001, respectively.

Since both the steroids or cAMP analog components of the EPC treatment to induce decidualization in this study could be upstream regulators of lncRNA expression, we determined the effects of vehicle, steroids (estradiol plus progesterone), cAMP analog, and EPC on the expression of the nine lncRNA genes. For *AC027288.3*, both steroids (*p* < 0.01) and cAMP (*p* < 0.001) caused significant 3.4- and 9.5-fold increases in expression (Figure 5A) compared to vehicle-treated cells, respectively. Incubation of cells with cAMP analog but not steroids caused a significant increase in the expression of AC108861.3, LINC02432, AC023154.1, BASP1-AS1, and LINC02600 compared to vehicle-treated cells (Figure 5B–F). However, in the presence of cAMP analog, steroids caused a significant increase in the expression of all five lncRNAs., indicating a cAMP-dependent effect of the steroids. The expression of *LINC02593* significantly (*p* < 0.001) increased in responses to cAMP analog compared to vehicle-treated cells (Figure 5G), while steroids had no effect. Both steroids and cAMP caused significant decreases in *LINC01605 and AL121578.3* expression compared to vehicle-treated cells, with the effect of the cAMP analog being much more pronounced for LINC01605 (Figure 5H–I).

### 3.5. PGR and FOXO1 as Regulators of LncRNA Expression

**Figure 6:**
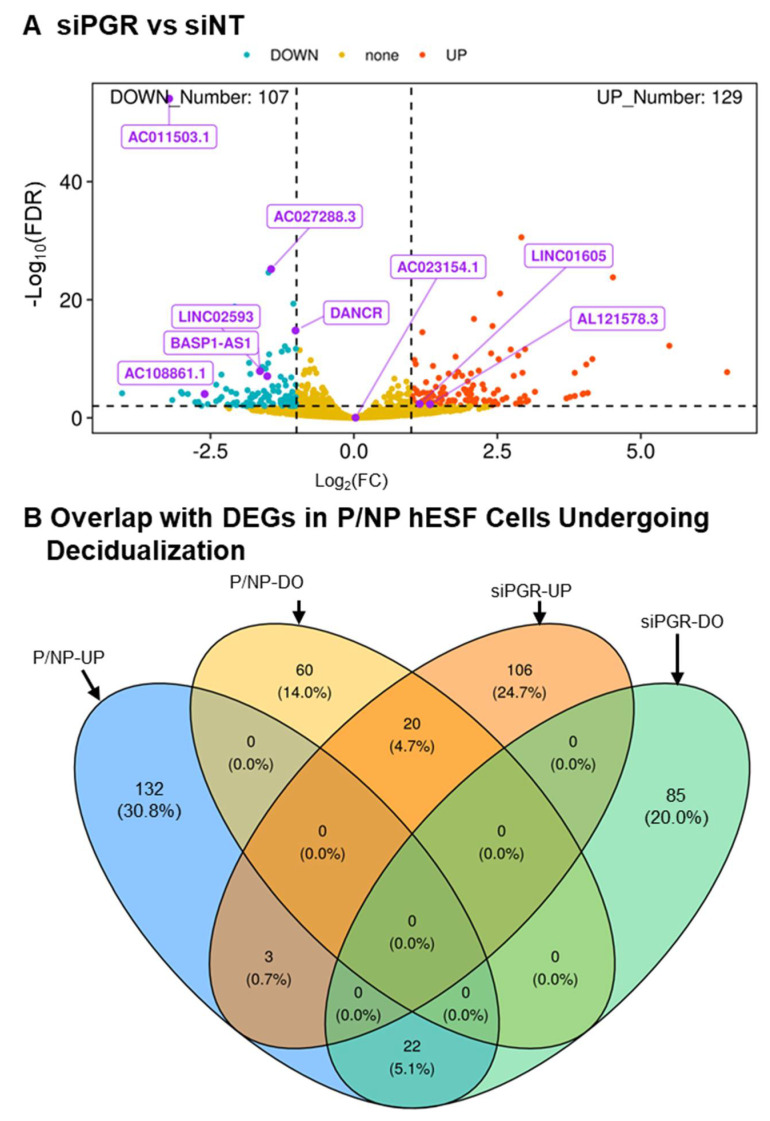
RNA-seq results of lncRNA genes regulated by PGR in non-pregnant (NP) hESF cells undergoing EPC-induced decidualization. (**A**) Volcano plot of reanalyzed RNA-seq results of NP hESF incubated with non-targeting (siNT) and PGR-targeting (siPGR) siRNA for two days, then EPC for three days. Abbrev: FDR, false-discovery rate; FC, fold change siPGR- compared to siNT-treated cells; NC, no change; UP increases in response to siPGR; DO, decreases in response to siPGR. Data are provided in Supplemental Tables S7 and S8. (**B**) Venn diagram comparing the RNA-seq results of the results in panel A to the common lncRNA’s differentially expressed in NP plus pregnant (P) hESF cells undergoing decidualization (see Figure 2B and Supplemental Table S6). Abbrev: P/NP-UP and -DO, common lncRNA genes between pregnant and non-pregnant hESF cells that increase and decrease during decidualization; NPsiPGR-UP and -DO, lncRNA’s upregulated and downregulated in response to siPGR, respectively.

**Figure 7:**
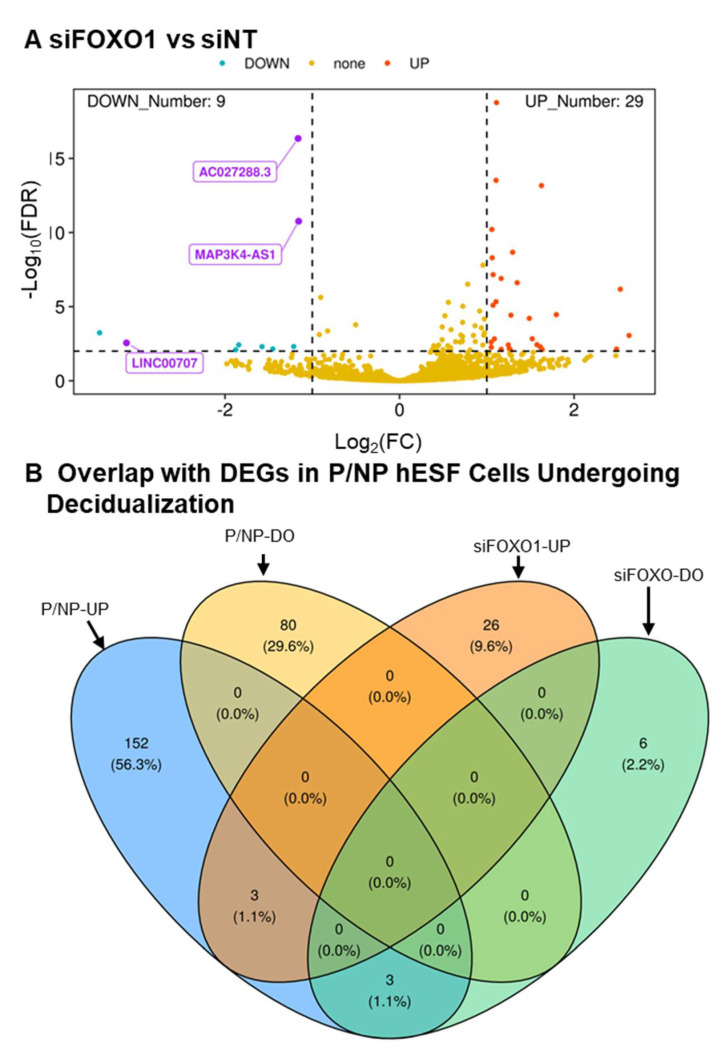
RNA-seq results of lncRNA genes regulated by FOXO1 in hESF cells undergoing EPC-induced decidualization. (**A**) Volcano plot of reanalyzed RNA-seq results of non-pregnant (NP) hESF incubated with non-targeting (siNT) and PGR-targeting (siPGR) siRNA for two days, then EPC for three days. Abbrev: FDR, false-discovery rate; FC, fold change sFOXO1- compared to siNT-treated cells; NC, no change; UP increases in response to siFOXO1; DO, decreases in response to siFOXO1. Data are provided in Supplemental Tables S9 and S10. (**B**) Venn diagram comparing the RNA-seq results of the results in panel A to the common lncRNA’s differentially expressed in NP plus pregnant (P) hESF cells undergoing decidualization (see Figure 2B and Supplemental Table S6). Abbrev: P/NP-UP and -DO, common lncRNA genes between pregnant and non-pregnant hESF cells that increase and decrease during decidualization; NPsiFOXO1-UP and -DO, lncRNAs upregulated and downregulated in response to siFOXO1, respectively.

PGR and FOXO1 are critical transcription factors that play vital roles in hESF decidualization [37,55]. Since little is known about what controls the differential expression of the lncRNA genes, we reanalyzed previously published RNA-seq data where cells were incubated with non-targeting (siNT) and transcription factor targeting (siPGR and siFOXO1) siRNAs for two days, followed by EPC for three days [32,33]. The result of the analysis for targeting PGR expression (Supplemental Table S8) is summarized in the volcano plot in Figure 6A. Differential expression was compared to the consensus differentially expressed genes in pregnant relative and non-pregnant hESF cells undergoing decidualization (Supplemental Table S6). As shown in the Venn diagram in Figure 6B (see Supplemental Table S9 for gene lists), the expression of 22 genes that increased during decidualization was downregulated with siPGR treatment. On the other hand, the expression of 20 genes that decreased during decidualization was downregulated with siPGR treatment. Six of these 42 PGR-regulated genes include *AC027288.3*, *AC108861.1*, *BASP1-AS1*, *LINC02593*, *AL121578.3* and *LINC01605*. The result of the analysis for targetting *FOXO1* expression (Supplemental Table S10) is summarized in the volcano plot in Figure 7A. Differential expression in response to targetting *FOXO1* expression was compared to the consensus differentially expressed genes in pregnant and non-pregnant hESF cells undergoing decidualization (Supplemental Table S6). As shown in the Venn diagram in Figure 7B (see Supplemental Table S11 for gene lists), the expression of 3 genes that increased during decidualization was downregulated with siFOXO1 treatment. On the other hand, the expression of none of the genes that decreased during decidualization was upregulated with siFOXO1 treatment. Interestingly, one of these FOXO1-regulated genes included *AC027288.3.* Therefore, the *AC027288.3* expression appears to be regulated by both PGR and FOXO1 during EPC-induced decidualization of hESF cells in vitro.

### 3.6. Potential Roles of Specific Transcription Factors on AC027288.3 Expression

**Figure 8:**
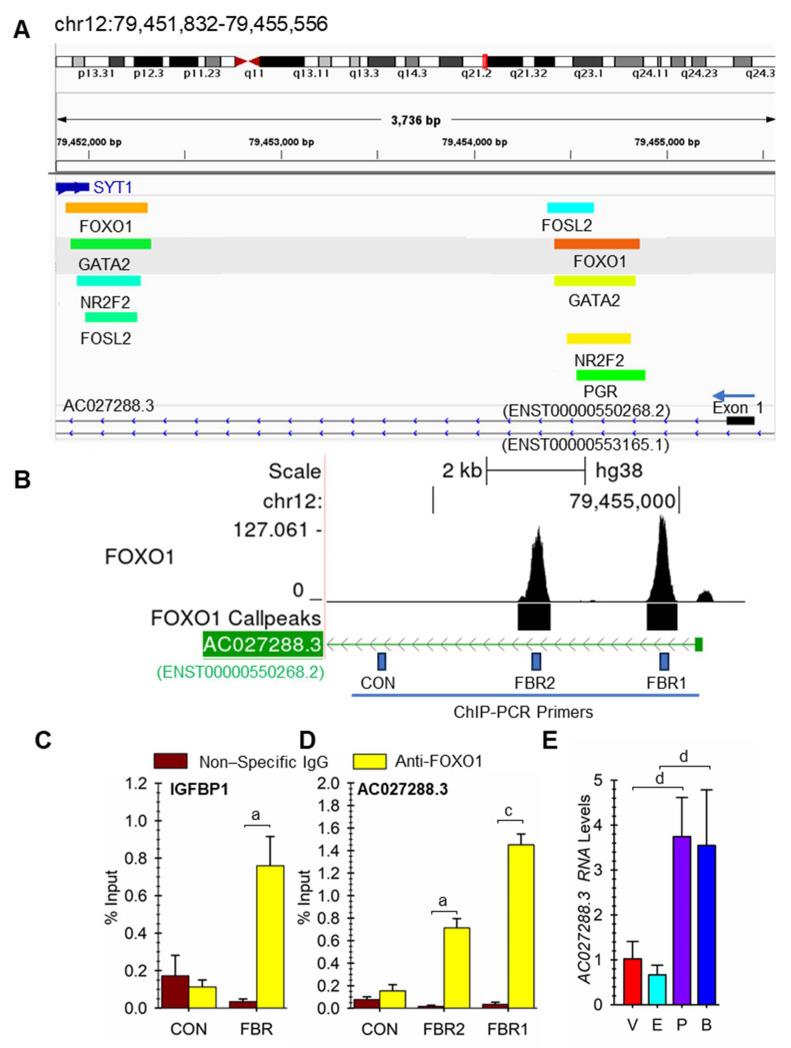
*FOXO1* and progesterone/PGR may directly regulate *AC027288.3* expression during hESF decidualization. (**A**) Map of select transcription factor binding sites near the ENST00000550268.2 transcript of the *AC027288.3* gene during hESF decidualization from previous ChIP-seq studies downloaded from ChIP-Atlas. A Color gradient represents −10*Log_10_(MACS2 Q-value) with blue (50), cyan (250), green (500), yellow (750), and red (>1000). (**B**) Map of FOXO1-binding sites near the *ENST00000550268.2* transcript of the *AC027288.3* gene during hESF decidualization from reanalysis of a previous ChIP-seq study. FBR1 and FBR2 represent the FOXO1-binding region primer amplicon region used in ChIP-PCR, while CON represents a control region ~3 kb downstream of FBR2. The picture was taken from the UCSC Genome Browser (http://genome.ucsc.edu). (**C**) ChIP-PCR of a well-known FOXO binding region near the IGFBP1 gene region. (**D**) ChIP-PCR results for FOXO1 binding to the FBR1 and FBR2 in the first intron of *ENST00000550268.2* near the transcriptional start site. Bars represent mean ± SEM using cells from four independent patients (N = 4). (**E**) Effects of vehicle (V), estradiol (E) versus progesterone (P), or both estradiol plus progesterone (B) on *AC027288.3* expression. Bars represent mean ± SEM using cells from six independent patients (N = 6). Statistical significance denoted as a, c, and d represents P less than 0.05, 0.005, and 0.001, respectively.

Several transcription factors such as FOSL2, FOXO1, GATA2, NR2F2, NCOA2, PGR, and ZBTB16 are known to play critical roles in non-pregnant hESF cells undergoing decidualization based on several cistrome studies [32,33,45,46,47,48]. As shown in Figure 8A, there appears to be a cluster of binding sites for FOSL2, FOXO1, GATA2, NR2F2, and PGR in the first intron near the first exon of *AC027288.3* (ENST00000550268.2) during hESF decidualization. A further downstream cluster within the same intron includes binding regions for the same transcription factors (Supplemental Figure S10). Focusing on the first cluster, we designed ChIP PCR primers for the two putative FOXO1 binding regions (FBR1 and FBR2) and a control (CON) region ~ 3 kb downstream from FBR2 (Figure 8B) to confirm FOXO1 binding in our hESF cells isolated from pregnant patients. As a positive control, we confirmed the enrichment of the binding of FOXO1 in our cells to a well-known region near the *IGFBP1* gene (Figure 8C), as previously published [32]. We were also able to confirm the enrichment of binding of FOXO1 in our cells to FBR1 and FBR2 of the first intronic region of *AC027288.3*, but not at a control region ~3 kb upstream of FBR2 (Figure 8D).

**Figure 9:**
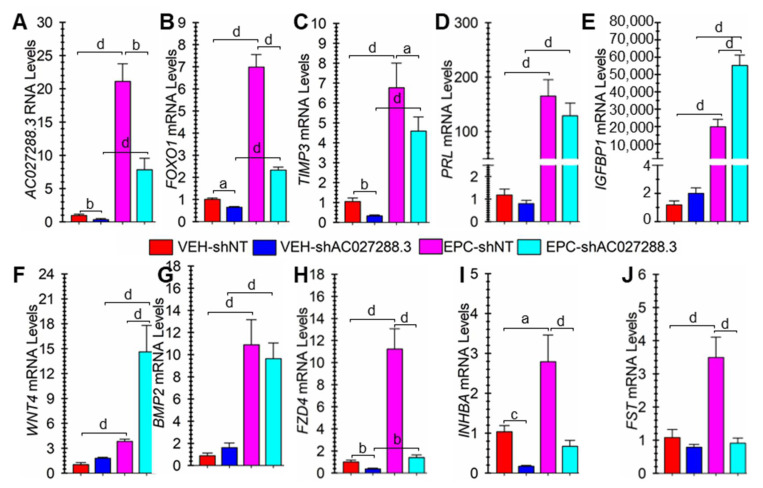
Targeting *AC027288.3* expression appears to affect decidualization. Cells were transduced with non-targeting (shNT) or *AC027288.3*-targeting (shAC027288.3) short hairpin RNA (shRNA) lentivirus. Then, they were treated for two days with vehicle (VEH) or stimulated to undergo decidualization with steroids plus cyclic adenosine monophosphate (cAMP) analog (EPC). (**A**) Relative *AC027288.3* RNA levels. Relative mRNA levels for the decidualization markers (**B**) *FOXO1*, (**C**) *IGFBP1*, (**D**) *PRL*, (**E**) *TIMP3*, (**F**) *WNT4*, (**G**) FZD4, (**H**) *BMP2*, (**I**) *INHBA*, and (**J**) *FST* were also determined. Graph bars represent mean ± SEM using cells from 6 independent patients (N = 6). Statistical significance denoted as a, b, and d represents P less than 0.05, 0.01, and 0.001, respectively.

Above, we showed that the combination of estradiol plus progesterone causes an increase in AC027288.3 expression (Figure 5A). Given that there are PGR binding sites in the first intron of AC027288.3 (Figure 9A), we hypothesized that the effect of steroid-induced expression was due to the actions of progesterone and not estradiol. To confirm this, we incubated the cells with the steroids individually and found that induction of *AC027288.3* expression is mediated by progesterone alone (Figure 8E).

### 3.7. Targeting AC027288.3 Expression Affects hESF Decidualization

Compared to non-targeting shRNA (shNT) lentivirus, transduction with AC027288.3-targeting (shAC027288.3) lentivirus caused a significant (*p* < 0.01) decrease in *AC023288.3* expression both in the absence or presence of EPC (Figure 9A). Targetting *AC027288.3* expression resulted in significantly lower expression of the classical decidualization markers *FOXO1* (*p* < 0.001; Figure 9B) and *TIMP3* (*p* < 0.05; Figure 9C) in EPC-treated cells. In addition, targeting *AC027288.3* expression resulted in significantly lower mRNA expression of *FOXO1* (*p* < 0.05) and *TIMP3* (*p* < 0.01) in vehicle-treated cells. Targeting *AC027288.3* expression did not affect *PRL* mRNA expression (Figure 9D). Alternatively, targeting *AC027288.3* expression resulted in significantly greater (*p* < 0.001) *IGFBP1* mRNA expression in EPC-treated cells (Figure 9E). A well-known BMP2-WNT:FZD-CTNBB1 pathway is known to control *FOXO1* expression during decidualization [56]. However, targeting *AC027288.3* expression caused a significant (*p* < 0.001) increase in WNT4 mRNA expression in EPC-treated cells (Figure 9F). On the other hand, targeting *AC027288.3* expression did not affect *BMP2* expression (Figure 9G). FZD4 is one of several frizzled receptors likely involved in WNT-mediated control of *FOXO1* expression during decidualization [56]. Targetting *AC027288.3* expression resulted in significantly lower expression of *FZD4* in vehicle- (*p* < 0.01) and EPC-treated (*p* < 0.001) cells (Figure 9H). Activin is critical in hESF decidualization [57] and negatively regulated by FST [58]. Interestingly, targeting AC027288.3 expression caused a significant decrease in *INHBB* mRNA expression (Figure 9I). On the other hand, it significantly (*p* < 0.001) prevented the increase in *FST* expression in the EPC-treated cells (Figure 9J).

## 4. Discussion

In the present study, we conducted a polyA+ RNA-Seq analysis of hESF cells from pregnant patients undergoing EP-induced decidualization in vitro. We compared this analysis to previously published data from hESF cells from non-pregnant patients under similar conditions. We found 158 and 82 common lncRNA genes whose expression is upregulated and downregulated during EPC-induced decidualization between the pregnant and non-pregnant hESF cell RNA-seq data. These genes included some, but not all, previously identified genes shown by other studies to be differentially expressed and play a function during hESF decidualization. Previously, we found that HAND2 plays a crucial role in hESF decidualization n [59], which was subsequently confirmed [60]. The data of our current study confirms a previous finding that the expression of *HAND2-AS1* is upregulated during hESF decidualization [18]. This increased expression has previously been shown to play a role in regulating *HAND2* expression [18]. A second lncRNA identified in this study that increased during hESF decidualization was *DANCR*. To our knowledge, the function of *DANCR* in hESF decidualization has not been reported. However, the role of *DANCR* as a cytoplasmic lncRNA that “sponges” particular microRNAs or interacts with various proteins has been described in other cell types [61,62]. Finally, *TUNAR* is known to decrease during hESF decidualization in vitro, and overexpressing it can negatively impact decidualization [21]. Interestingly, *TUNAR* expression was not detected as common differentially expressed in this study. Notably, one caveat of our analysis is it would not include any non-polyadenylated lncRNAs since the RNA-seq libraries were constructed with poly A+ RNA.

*AC027288.3* lncRNA appears to be an upstream regulator of FOXO1 expression during hESF decidualization. FOXO1 is a transcription factor whose expression is commonly used as a decidualization marker and is also known to play a vital role in decidualization [32,37,55,63,64,65]. FOXO1 controls the expression of several downstream target genes, such as *IFBP1*, *PRL*, and *TIMP3*. Targetting *AC027288.3* expression in hESF cells in our current study resulted in a marked downregulation of *FOXO1* expression during decidualization. *A* BMP2-WNT/FZD-CTNBB1 pathway regulates *FOXO1* expression during hESF decidualization, and BMP2 also controls *FZD4* expression [56]. In our current study, targeting AC027288.3 expression during hESF decidualization did not influence *BMP2* expression but significantly increased *WNT4* and decreased *FZD4* expression. Therefore, it may play a role in controlling *FZD4* expression, which may be why *FOXO1* expression decreased by targeting the expression of *AC027288.3*. Even though *WNT4* expression is increased, the decreased expression of *FZD4* with *AC027288.3* knockdown may lead to a resulting decrease in the BMP-WNT/FZD-CTNBB1 pathway signaling, which may have led to the decreased *FOXO1* expression. Additional studies are needed to evaluate this.

Besides BMPs, activins are another set of TGFβ receptor ligands known to play a role in hESF decidualization. Human ESF cells show increased *INHBA* expression at the onset of decidualization in vivo and in vitro [66,67,68]. Treatment of hESF cells with activins enhances the decidualization of hESF cells in vitro [57]. Surprisingly, *INHBA* expression was dramatically reduced during decidualization when *AC027288.3* expression was targeted in this study. FST is an inhibitor of activin signaling by binding to the ligand and preventing the binding and activation of their receptors [58]. Our results indicated *FST* expression increases during hESF decidualization, and targetting *AC027288.3* expression prevented increases in FST expression. Strong evidence suggests that activin-SMAD2/3 signaling is crucial for normal decidualization in mice [69,70]. This is also possible for human decidualization [71]. The role of *AC027288.3* in regulating the activin-SMAD2/3 signaling hESF decidualization needs to be investigated further.

Two major regulators of hESF decidualization are PGR and cAMP [71]. Further, although progesterone alone can induce decidualization, part of the action of progesterone is to increase cAMP levels in the cells [72]. Thus, PGR and increased levels of cAMP can individually or synergistically regulate decidualization and play a role in its maintenance. Of the nine lncRNA genes whose expression was examined more closely in this study, steroids alone only slightly influenced the expression of one lncRNA (*AC027288.3*), which was found to be dependent on the action of progesterone alone. On the other hand, the addition of cAMP influenced the expression of all nine. Of these, five showed a cAMP analog-dependent effect of steroids, the most pronounced being for the expression of *AC027288.3*. Further, reanalysis of RNA-seq data revealed that 42 lncRNA genes are differentially expressed during hESF decidualization and likely controlled directly or indirectly by PGR. These results show that, similarly to protein-coding genes, the expression of differentially expressed lncRNA genes during hESF decidualization can involve the actions of both cAMP and progesterone-PGR signaling.

FOXO1 is a significant regulator of hESF decidualization [32,33]. In our current study, we found the expression of three lncRNA genes differentially expressed during hESF decidualization, which are likely controlled directly or indirectly by FOXO1. Indeed, FOXO1 ChIP-seq data from hESF cells undergoing decidualization revealed the binding of FOXO1 to regions of the first intron of *AC027288.3*. This FOXO1 binding was confirmed for some of these sites using ChIP-PC. These results seem confusing since the results of this study suggest that FOXO1 and AC027288.3 may be regulating each other’s expression in hESF cells undergoing decidualization. We speculate that this positive feedback might be involved in the commitment towards the decidual cell phenotype. Finally, previously published ChIP-seq data show that other transcription factors may also be involved in directly regulating *AC027288.3* expression during decidualization, including PGR, FOSL2, GATA2, and NR2F2. Each of these is also a key regulator of hESF decidualization [32,33,45,46,47,48]. Therefore, the differential expression of at least some of the lncRNA genes during hESF decidualization is likely directly orchestrated by the same transcription factors that control protein-coding genes during hESF decidualization.

Besides *AC027288.3*, the precise function of the other eight lncRNAs of Table 1 in decidualization remains to be determined. Besides differential expression in other cells/tissues, the biological functions for *AC023154.1*, *AC108861.1*, *AL121578.3*, and *LINC02593* lncRNAs do not currently appear in the literature. However, an interesting study reveals that during neuronal differentiation, *BASP1-AS1* lncRNA regulates the expression of its adjacent protein-coding gene *BASP1* [73]. Together, *BASP1-AS1* RNA and BASP1 protein form a molecular complex that also includes the ubiquitous bHLH protein TCF12 [73]. The ability of *BASP1-AS1* RNA to form a complex with TCF12 is interesting as several bHLH transcription factors play a key role in human endometrial decidualization [27,59,74,75,76,77,78,79,80,81,82,83]. Targetting *LINC01605* expression inhibits autophagy and induces apoptosis in Tenon’s capsule fibroblasts [84]. In addition, *LINC01605* RNA may promote aerobic glycolysis, proliferation, migration, and invasion of cancer cells [85,86]. However, the functional role for decreasing expression of *LINC01605* during hESF decidualization noted in our study remains to be determined. *LINC02432* lncRNA may regulate HK2 and ferroptosis in cancer [87], which may contribute to the evidence that decidualization involves metabolic reprogramming and an increased reliance on aerobic glycolysis [83,88]. Finally, it appears a bi-directional promotor possibly controls *LINC02600* expression as its 5′-end is very close to the 5′-end of the protein-coding gene *ADRA2C*. The increased expression and function of *ADRA2C* in decidualization have been studied [89], but the role of *LINC02600* lncRNA in regulating its expression during decidualization remains to be determined.

Collectively, our results provide the identity of several new lncRNA genes that are differentially expressed and may play a role in hESF decidualization. The results of this study further support the hypothesis that one lncRNA, AC027288.3, may play a role in hESF decidualization by regulating *FZD4* and *INHBA* expression. In addition, our results suggest *AC027288.3* expression during hESF decidualization could be regulated using several transcription factors, including PGR, FOXO1, GATA2, NR2F2, and FOSL2. Additional experiments are warranted to determine the precise function and control of the expression of AC027288.3, and other lncRNAs demonstrated to be differentially expressed during hESF decidualization in this study.

## Figures and Tables

**Table 1:** DNA oligonucleotide sequences (5′- to 3′-prime) used for RT-qPCR in this study.

Gene	Upstream	Downstream
*AC023154.1*	AAGCCTTCTGGAGGAGAAGCA	TGGTGGCTCAGCAACATCTA
*AC027288.3*	GCAGCTTGGCAATGAAGTCA	CTCTGTCAGCCTCCCTCTTC
*BMP2*	CAGACCACCGGTTGGAGA	CCACTCGTTTCTGGTAGTTCTTC
*AC108861.1*	AGAAGCGTGCGTGCTACA	TTTGAAGCTGTCTGTGCAGTTG
*AL121578.3*	ACCTTCACATGTCCGAATGC	TCCTGTCCCCACCTCTAAGA
*BASP1-AS1*	AGCACCGGGACACAGAATAG	TTTGCGGGAAGGTAAAATTG
*DANCR*	CTGCATTCCTGAACCGTTATCT	GGGTGTAATCCACGTTTCTCAT
*FOXO1*	CTGGCTCTCACAGCAATGAT	CACCATAGAATGCACATCCC
*FZD4*	AACTTTCACACCGCTCATCC	TGCACATTGGCACATAAACA
*IGFBP1*	GCTCTCCATGTCACCAACAT	TCTCCTGATGTCTCCTGTGC
*H36B4*	GACACCCTCCAGGAAGCGA	GTGTTCGACAATGGCAGCAT
*LEFTY2*	CCTGAGAGGGTGCTAAGAG	GGTAGGTAGGGGCTGTCT
*LINC01605*	TTCCCGTTACAAACAGCCGA	ACTGCCTCTGTCTCCTGTCA
*LINC02432*	ATGCTGTGGAGGCTCTGTTG	AAAAGCAGTGTGGACCCGAA
*LINC02593*	TCCAAGTCCAGTCTGTCCAC	AGCATCCCTGACTACACACC
*LINC02600*	CAGGAGGAGCTGAGGATGAG	GGACGTCACTCTCCAAAGGA
*MALAT1*	GAATTGCGTCATTTAAAGCCTAGTT	GTTTCATCCTACCACTCCCAATTAAT
*PGR*	TCGCCTTAGAAAGTGCTGTC	GCTTGGCTTTCATTTGGAACG
*PRL*	AGCCAGGTTCATCCTGAAA	AGCAGAAAGGCGAGACTCTT
*TIMP3*	TGACAGGTCGCGTCTATGAT	CAACCCAGGTGATACCGATAG
*WNT4*	AGCCCTCATGAACCTCCAC	CACCCGCATGTGTGTCAG
*XIST*	ACGCTGCATGTGTCCTTAG	GAGCCTCTTATAGCTGTTTG

**Table 2:** DNA oligonucleotide sequences (5′- to 3′-prime) that were used for ChIP-qPCR in this study.

Near Gene (Site)	Upstream	Downstream
AC027288.3(FBR1)	GCCTGACTCTGGTGGTAAGC	TGCTGGCATTCAGTGCAGTT
AC027288.3(FBR2)	CAGTTACCTTCCACCCCGAC	GGTAGAGATTTGTGTGCAGCC
AC027288.3(CON)	AGGACGCTTTATAGTCGGGC	TGGCATCGTTCTTGCCAATC
IGFBP (CON)	AGATAGGGATTGGTTCGCGTAT	GGTCTGTGCTAACAATGCCAC
IGFBP1 (FBR)	TTCAGAGCATGGATTGCCCA	CTTCCCAATCCTGCCTTCGT

## Data Availability

The raw RNA-seq data presented in the study are openly available in NCBI as project PRJNA368634. Other data are available upon reasonable request to the corresponding author.

## Supplementary Materials

The following supporting information can be downloaded at: https://www.mdpi.com/article/10.3390/cells13090778/s1, Supplemental Table Legends. Table S1: RNA-seq results of protein-coding genes in hESF cells isolated from pregnant patients treated with VEH (not undergoing decidualization) or EPC (undergoing decidualization). FDR, false-discovery rate; Log2FC, log (base 2) Fold-Change (EPC vs. VEH). Cells were used from three independent patients (N = 3). Table S2: RNA-seq results of protein-coding genes in hESF cells isolated from non-pregnant patients treated with VEH (not undergoing decidualization) or EPC (undergoing decidualization). FDR, false-discovery rate; Log2FC, log base 2 Fold-Change (EPC vs. VEH). Cells were used from three independent patients (N = 3). Table S3: Combined RNA-seq results of protein-coding genes from hESF cells isolated from both pregnant and non-pregnant patients treated with VEH (not undergoing decidualization) or EPC (undergoing decidualization). See Figure 1B. Table S4: RNA-seq results of lncRNA genes in hESF cells isolated from pregnant patients treated with VEH (not undergoing decidualization) or EPC (undergoing decidualization). FDR, false-discovery rate; Log2FC, log base 2 Fold-Change (EPC vs. VEH). Cells were used from three independent patients (N = 3). Table S5: RNA-seq results of lncRNA genes in hESF cells isolated from non-pregnant patients treated with VEH (not undergoing decidualization) or EPC (undergoing decidualization). FDR, false-discovery rate; Log2FC, log base 2 Fold-Change (EPC vs. VEH). Cells were used from three independent patients (N = 3). Table S6: Combined RNA-seq results of lncRNA genes from hESF cells isolated from both pregnant and non-pregnant patients treated with VEH (not undergoing decidualization) or EPC (undergoing decidualization). See Figure 2B. Table S7: Information on the nine lncRNA genes of interest, including their Symbol, ENSEMBL GeneID, TranscriptIDs, and primary transcripts (or exons, as determined in Supplemental Figures S1–S9). Table S8: lncRNA genes regulated by PGR during decidualization. RNA-seq results of lncRNA genes in hESF cells isolated from non-pregnant patients treated with siNT or siPGR for two days, followed by EPC for three days. FDR, false-discovery rate; Log2FC, log base 2 Fold-Change (EPC vs. VEH). Cells were used from three independent patients (N = 3). Table S9: Combined RNA-seq results of lncRNA genes differentially expressed during decidualization (common between hESF cells isolated from both pregnant and non-pregnant patients) plus regulated by PGR. See Figure 6B. Table S10: lncRNA genes regulated by FOXO1 during decidualization. RNA-seq results of lncRNA genes in hESF cells isolated from non-pregnant patients treated with siNT or siFOXO1 for two days, followed by EPC for three days. FDR, false-discovery rate; Log2FC, log base 2 Fold-Change (EPC vs. VEH). Cells were used from three independent patients (N = 3). Table S11: Combined RNA-seq results of lncRNA genes differentially expressed during decidualization (common between hESF cells isolated from both pregnant and non-pregnant patients) plus regulated by FOXO1. See Figure 7B. Supplemental Figures. Figure S1. RNA-Seq results at the *AC027288.3* (ENSG00000257894) gene region on chromosome 12. Three transcripts (in green) are associated with this gene, and only one (ENST00000550268.2) expression is upregulated in EPC-treated cells compared to VEH-treated cells. The other transcripts do not seem to be expressed in the samples. This transcript is antisense to the *SYT1* gene with no overlapping exonic regions. The picture was taken from the UCSC Genome Browser (http://genome.ucsc.edu). Figure S2. RNA-Seq results at the *AC023154.1* (ENSG00000248115) gene region on chromosome 4. Three transcripts (in green) are associated with this gene, and only one (ENST00000510351.1) expression is upregulated in EPC-treated cells compared to VEH-treated cells. The gene is antisense to an intron region of the protein-coding gene *SCFD2*. The picture was taken from the UCSC Genome Browser (http://genome.ucsc.edu). Figure S3. RNA-Seq results at the *AC108861.1* (ENSG00000278408) gene region on chromosome 15. One transcript (in green) is associated with this gene (ENST00000611634.1), and its expression is upregulated in EPC-treated cells compared to VEH-treated cells. The gene is antisense to the protein-coding *THSD4* and has four different protein-coding transcripts (not all shown), and one (ENST00000357769.4) has its first exon overlapping with the third exon of AC108861.1. The picture was taken from the UCSC Genome Browser (http://genome.ucsc.edu). Figure S4. RNA-Seq results at the *AL121578.3* (ENSG00000259977) gene region on chromosome X. One transcript (in green) is associated with this gene (ENST00000567273.1) and its expression is downregulated in EPC-treated compared to VEH-treated cells. The gene is antisense and overlaps with an intron of another gene, *ENSG00000250349*, which encodes a tetraspanin family member. The picture was taken from the UCSC Genome Browser (http://genome.ucsc.edu). Figure S5. RNA-Seq results at the *BASP1-AS1* gene region on chromosome 5. A. Five transcripts (in green) are associated with this gene on chromosome 5. It is antisense to *BASP1* (which has two alternate transcripts) and a close inspection of the first exons of *BASP1-AS1* and BASP1. B. The first exon of the main BASP1 transcript does not overlap with the first exon of the BASP1-AS1 transcripts. However, the first exon of a minor *BASP1* transcript overlaps with two *BASP1-AS1* transcripts (ENST00000399760.2, ENST00000667677.1). Although the expression of this gene is upregulated in EPC-treated cells compared to VEH-treated cells, it is unclear which of the five transcripts is involved. The picture was taken from the UCSC Genome Browser (http://genome.ucsc.edu). Figure S6. RNA-Seq results at the *LINC01605* gene region on chromosome 8. Four transcripts (in green) are associated with this gene, and two (ENST00000522718.5, ENST00000520422.5) appear to be more highly expressed in VEH-treated cells than EPC-treated cells undergoing decidualization. The picture was taken from the UCSC Genome Browser (http://genome.ucsc.edu). Figure S7. Table of information of the lncRNA genes of interest, including their Symbol, ENSEMBL GeneID, TranscriptIDs, and primary transcripts (or exons, as determined in Supplemental Figures S1–S9). RNA-Seq results at the *LINC02432* gene region on chromosome 4. Three transcripts (in green) are associated with this gene, and one (ENST00000701566.1) appears to be more highly expressed in EPC-treated cells than in VEH-treated cells undergoing decidualization. The picture was taken from the UCSC Genome Browser (http://genome.ucsc.edu). Figure S8. RNA-Seq results at the *LINC02593* gene region on chromosome 1. One transcript (in green) is associated with this gene and appears more highly expressed in EPC-treated cells than VEH-treated cells undergoing decidualization. The closest gene is approximately 3 kb away and is *SAMD11*, with their 5” ends facing each other. The 5′-end of *LINC02593* may be much closer to that of *SAMD11* than is currently annotated on the UCSC Genome Browser. The picture was taken from the UCSC Genome Browser (http://genome.ucsc.edu). Figure S9. RNA-Seq results at the *LINC02600* gene region on chromosome 4. One transcript (in green) is associated with this gene and appears more highly expressed in EPC-treated cells than VEH-treated cells undergoing decidualization. The closest gene is approximately 2 kb away and is *ADRA2C,* with their 5” ends facing each other. The 5′-ends of *LINC02600* and *ADRA2C* may be much closer than is currently annotated on the UCSC Genome Browser. The picture was taken from the UCSC Genome Browser (http://genome.ucsc.edu). Figure S10. Transcription factor binding site cluster 2 in the first intron of ENST00000550268.2 (AC027288.3) from multiple ChIP-seq publications. A Bed file containing the data were downloaded from Chip Atlas and displayed on the IGV genome browser. A Color gradient represents −10*Log_10_(MACS2 Q-value) with blue (50), cyan (250), green (500), yellow (750), and red (>1000).
