# Supplementary material for: The Long Non-Coding RNA Gene AC027288.3 Plays a Role in Human Endometrial Stromal Fibroblast Decidualization
Source: Cells. 2024 May 2;13(9):778. doi: 10.3390/cells13090778 (PMC11083667; doi:10.3390/cells13090778)
Supplement: Supplementary file 1 [file cells-13-00778-s001.zip › cells-2969671 - Supplemental Figures.pdf]

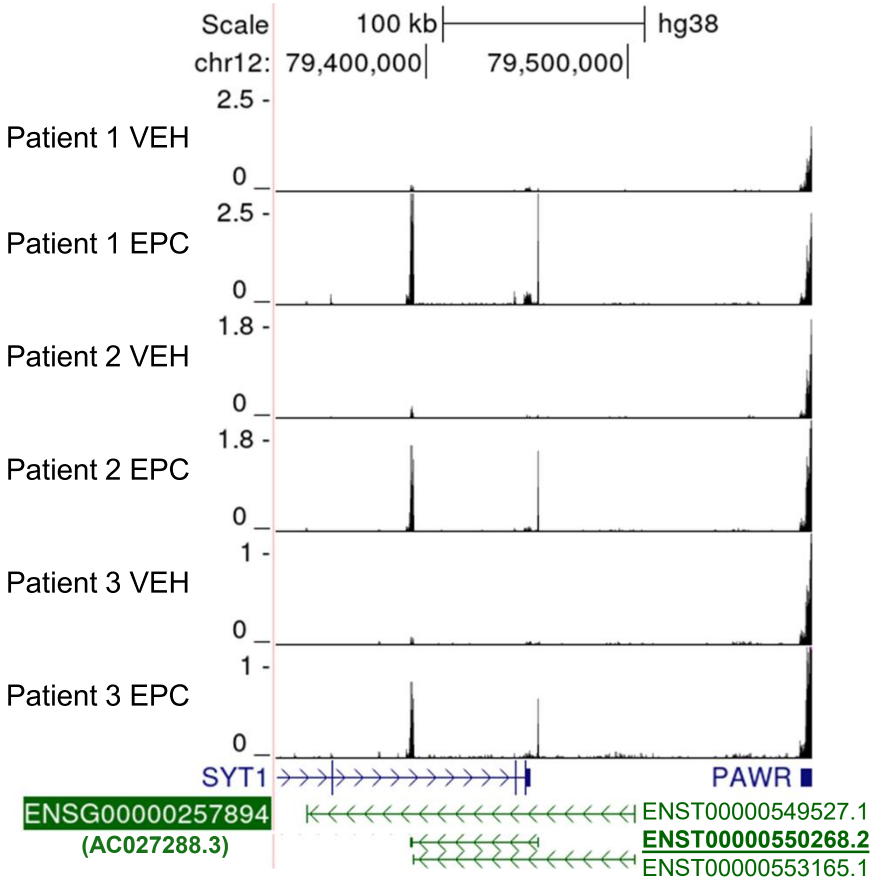

Figure S1. RNA-Seq results at the *AC027288.3* (ENSG00000257894) gene region on chromosome 12. Three transcripts (in green) are associated with this gene, and only one (ENST00000550268.2) expression is upregulated in EPC-treated cells compared to VEH-treated cells. The other transcripts do not seem to be expressed in the samples. This transcript is antisense to the *SYT1* gene with no overlapping exonic regions. The picture was taken from the UCSC Genome Browser (http://genome.ucsc.edu).

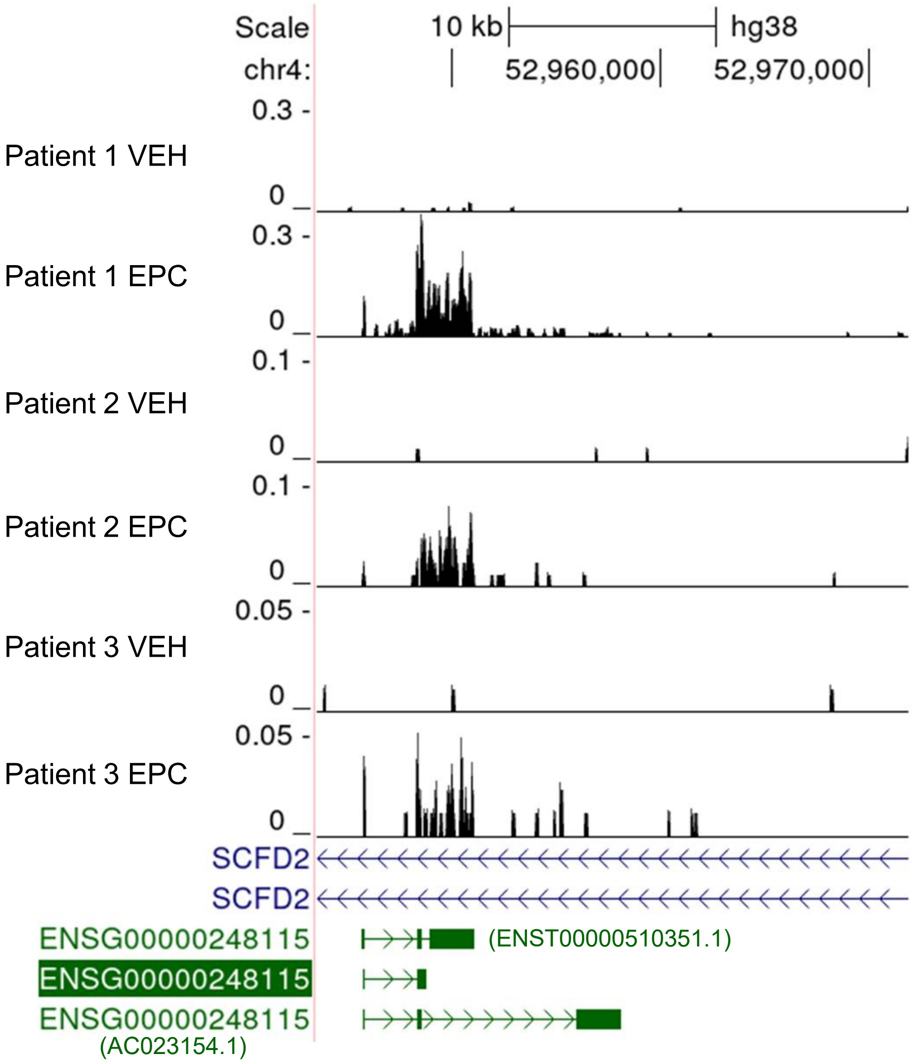

Figure S2. RNA-Seq results at the *AC023154.1* (ENSG00000248115) gene region on chromosome 4. Three transcripts (in green) are associated with this gene, and only one (ENST00000510351.1) expression is upregulated in EPC-treated cells compared to VEH-treated cells. The gene is antisense to an intron region of the protein-coding gene *SCFD2*. The picture was taken from the UCSC Genome Browser (http://genome.ucsc.edu).

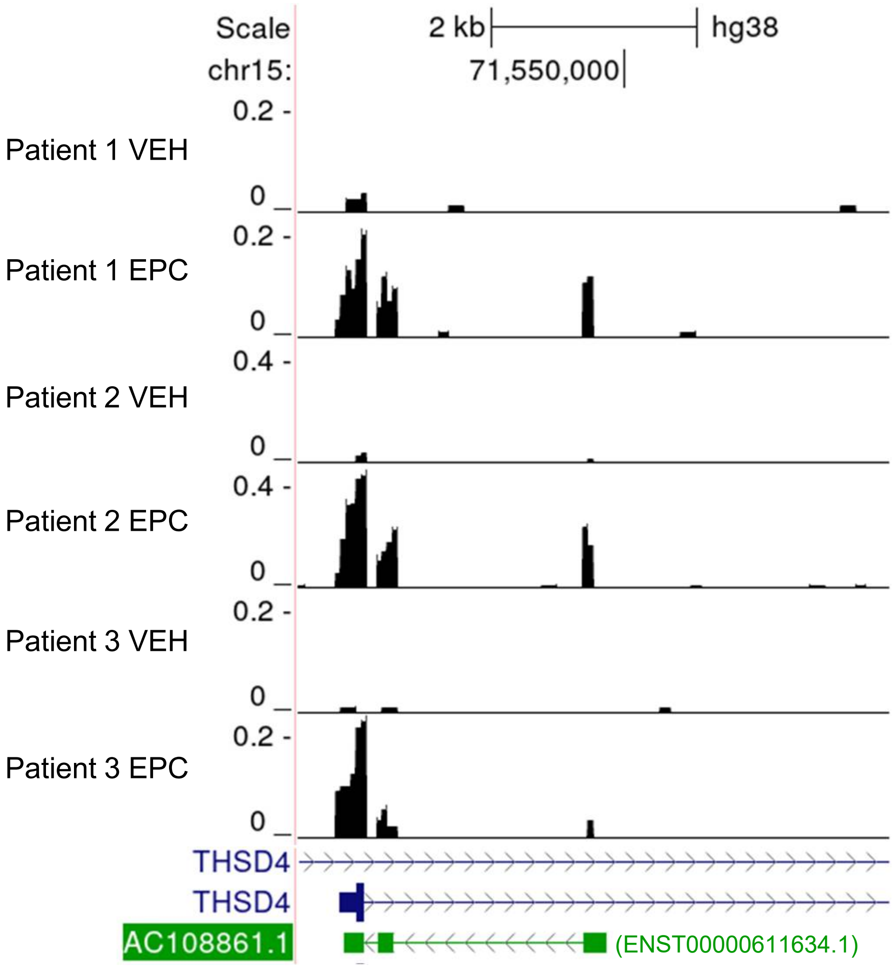

Figure S3. RNA-Seq results at the *AC108861.1* (ENSG00000278408) gene region on chromosome 15. One transcript (in green) is associated with this gene (ENST00000611634.1), and its expression is upregulated in EPC-treated cells compared to VEH-treated cells. The gene is antisense to the protein-coding *THSD4* and has four different protein-coding transcripts (not all shown), and one (ENST00000357769.4) has its first exon overlapping with the third exon of AC108861.1. The picture was taken from the UCSC Genome Browser (http://genome.ucsc.edu).

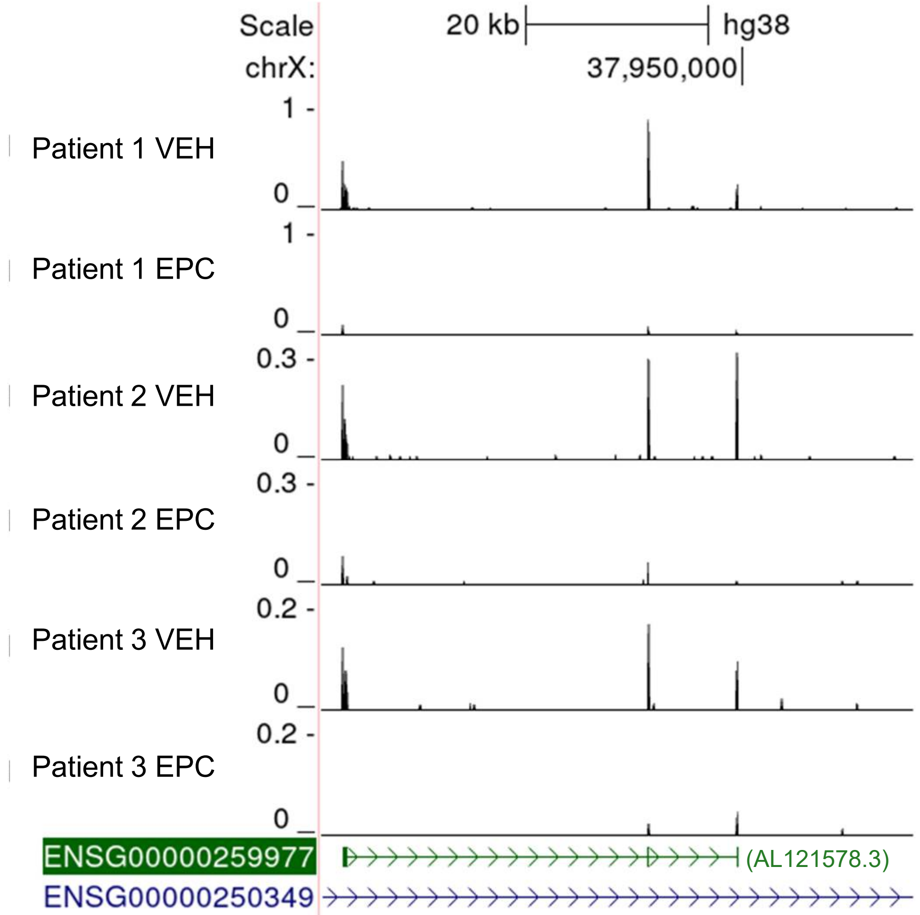

Figure S4. RNA-Seq results at the *AL121578.3* (ENSG00000259977) gene region on chromosome X. One transcript (in green) is associated with this gene (ENST00000567273.1) and its expression is downregulated in EPC-treated compared to VEH-treated cells. The gene is antisense and overlaps with an intron of another gene, *ENSG00000250349*, which encodes a tetraspanin family member. The picture was taken from the UCSC Genome Browser (http://genome.ucsc.edu).

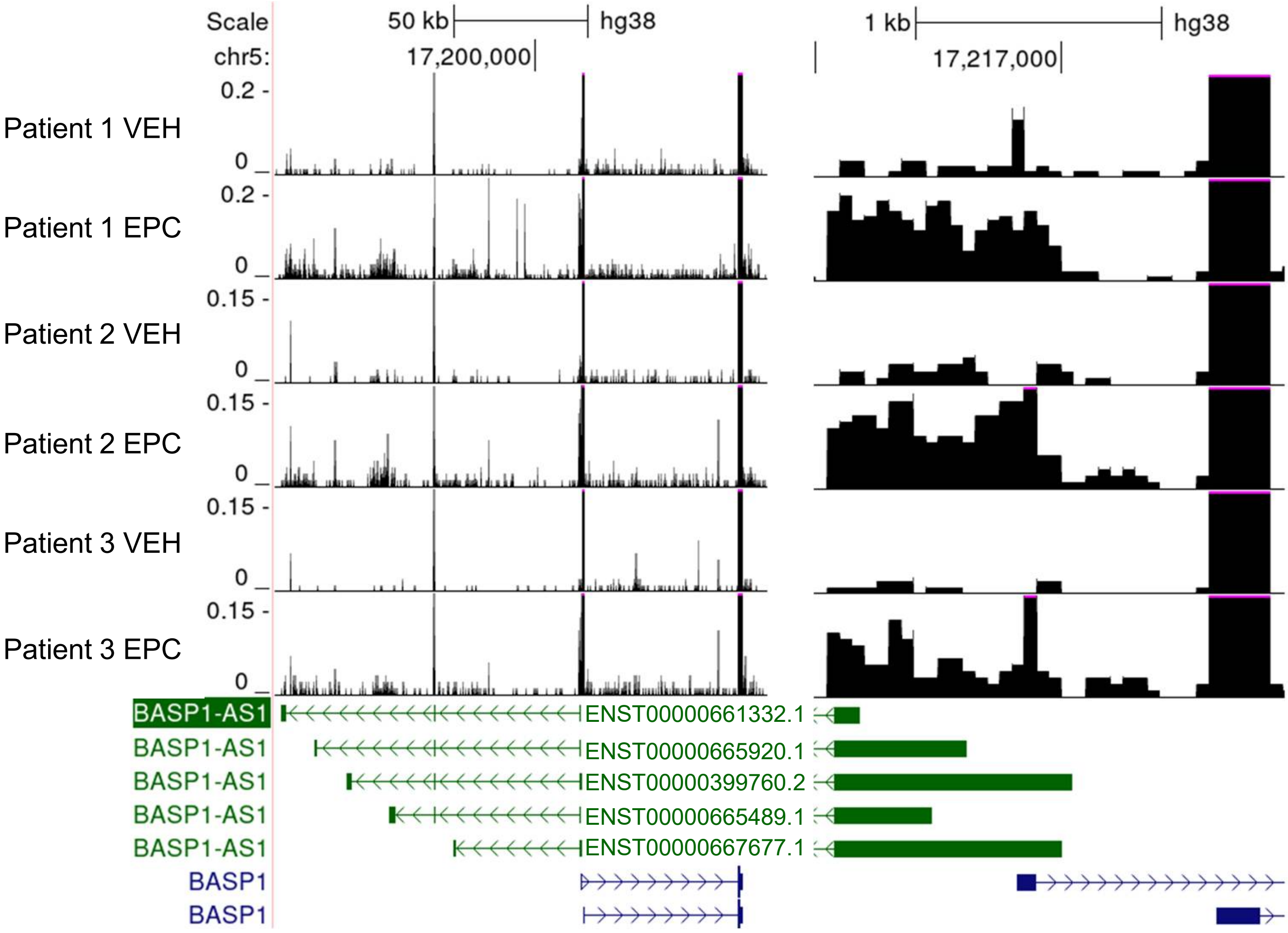

Figure S5. RNA-Seq results at the *BASP1-AS1* gene region on chromosome 5. A. Five transcripts (in green) are associated with this gene on chromosome 5. It is antisense to *BASP1* (which has two alternate transcripts) and a close inspection of the first exons of *BASP1-AS1* and BASP1. B. The first exon of the main BASP1 transcript does not overlap with the first exon of the BASP1-AS1 transcripts. However, the first exon of a minor *BASP1* transcript overlaps with two *BASP1-AS1* transcripts (ENST00000399760.2, ENST00000667677.1). Although the expression of this gene is upregulated in EPC-treated cells compared to VEH-treated cells, it is unclear which of the five transcripts is involved. The picture was taken from the UCSC Genome Browser (http://genome.ucsc.edu).

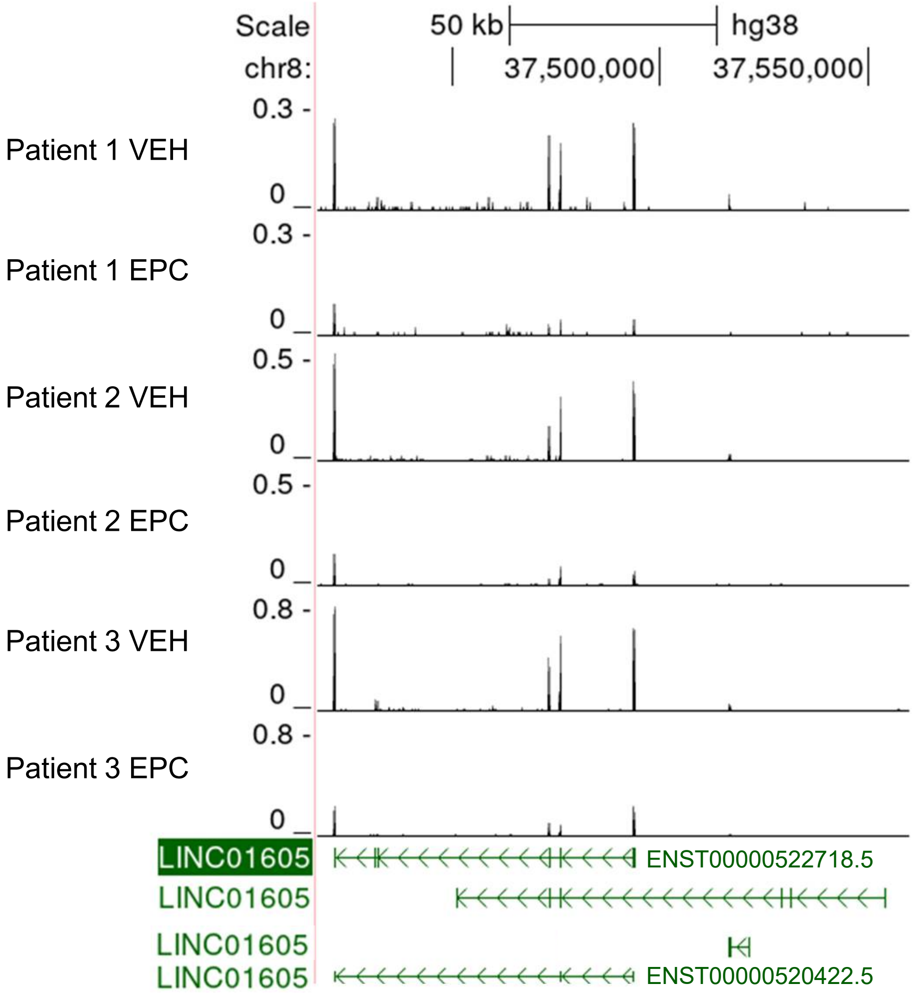

Figure S6. RNA-Seq results at the *LINC01605* gene region on chromosome 8. Four transcripts (in green) are associated with this gene, and two (ENST00000522718.5, ENST00000520422.5) appear to be more highly expressed in VEH-treated cells than EPC-treated cells undergoing decidualization. The picture was taken from the UCSC Genome Browser (http://genome.ucsc.edu).

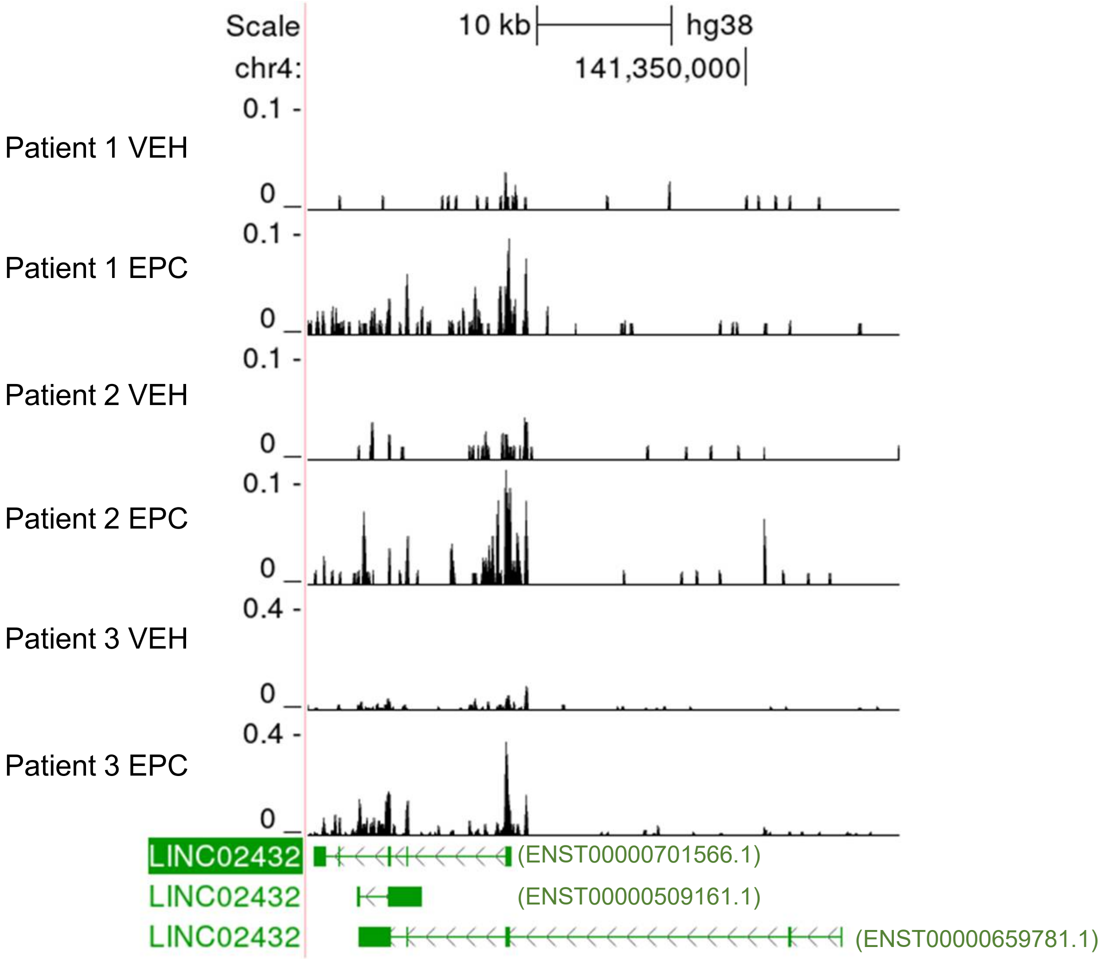

Figure S7. Table of information of the lncRNA genes of interest, including their Symbol, ENSEMBL GeneID, TranscriptIDs, and primary transcripts (or exons, as determined in Supplemental Figures S1–S9). RNA-Seq results at the *LINC02432* gene region on chromosome 4. Three transcripts (in green) are associated with this gene, and one (ENST00000701566.1) appears to be more highly expressed in EPC-treated cells than in VEH-treated cells undergoing decidualization. The picture was taken from the UCSC Genome Browser (http://genome.ucsc.edu).

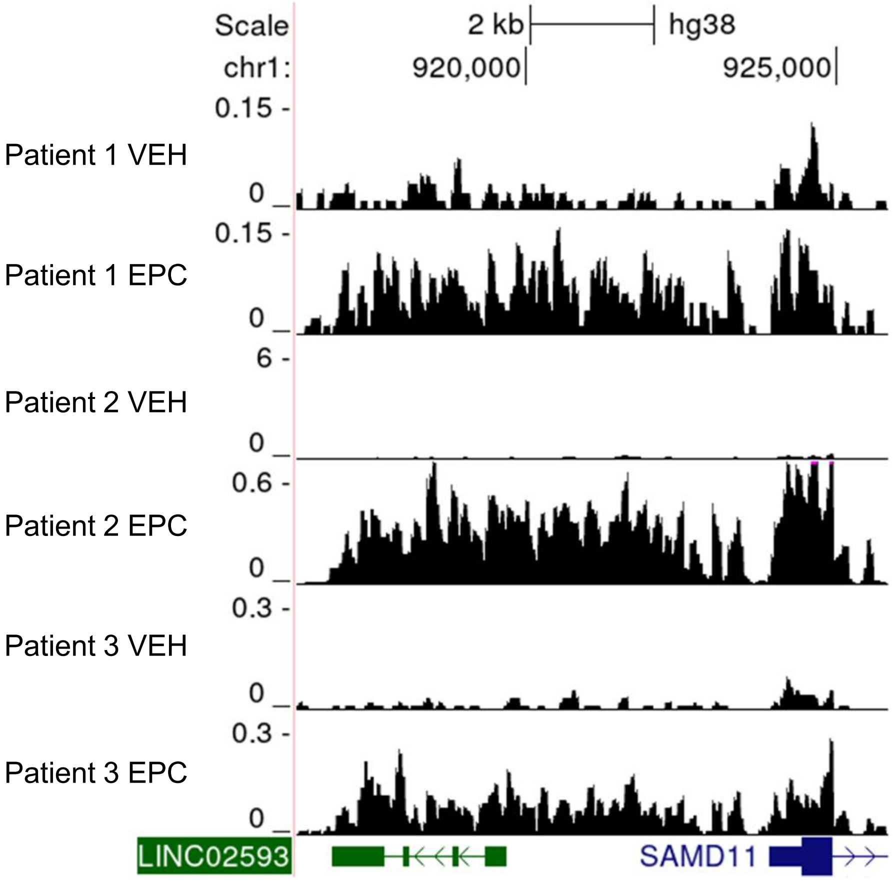

Figure S8. RNA-Seq results at the *LINC02593* gene region on chromosome 1. One transcript (in green) is associated with this gene and appears more highly expressed in EPC-treated cells than VEH-treated cells undergoing decidualization. The closest gene is approximately 3 kb away and is *SAMD11*, with their 5″ ends facing each other. The 5′-end of *LINC02593* may be much closer to that of *SAMD11* than is currently annotated on the UCSC Genome Browser. The picture was taken from the UCSC Genome Browser (http://genome.ucsc.edu).

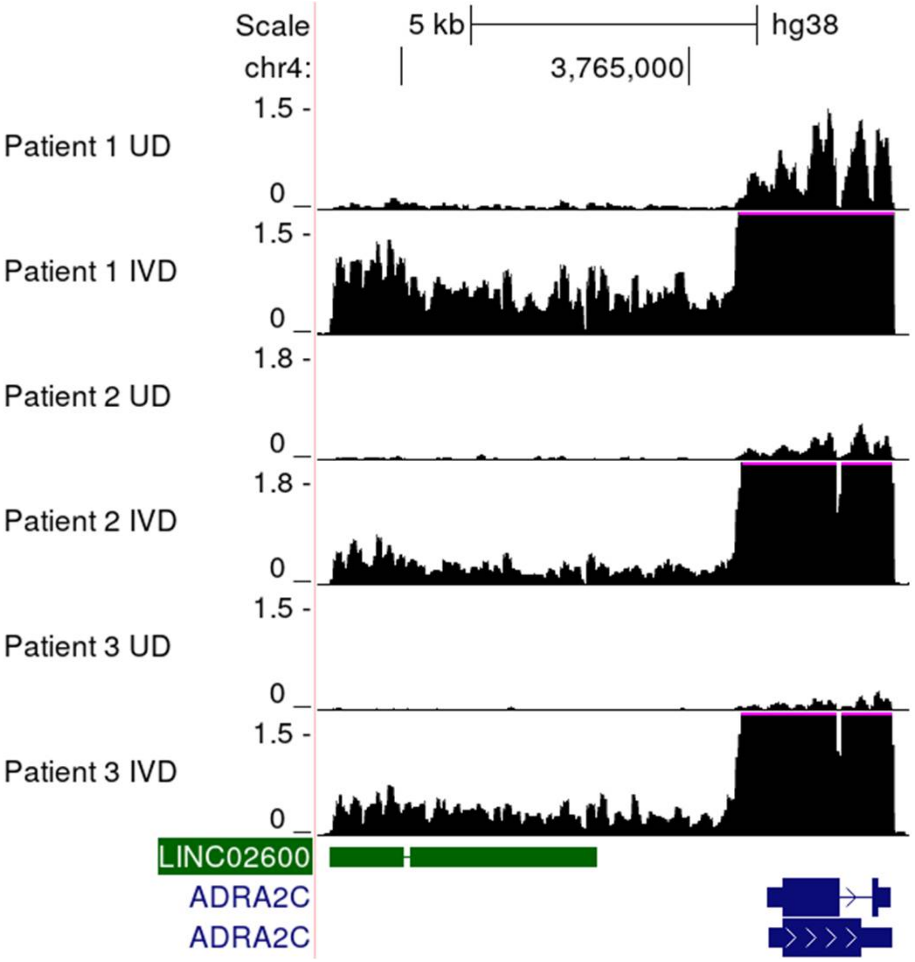

Figure S9. RNA-Seq results at the *LINC02600* gene region on chromosome 4. One transcript (in green) is associated with this gene and appears more highly expressed in EPC-treated cells than VEH-treated cells undergoing decidualization. The closest gene is approximately 2 kb away and is *ADRA2C,* with their 5″ ends facing each other. The 5′-ends of *LINC02600* and *ADRA2C* may be much closer than is currently annotated on the UCSC Genome Browser. The picture was taken from the UCSC Genome Browser (http://genome.ucsc.edu).

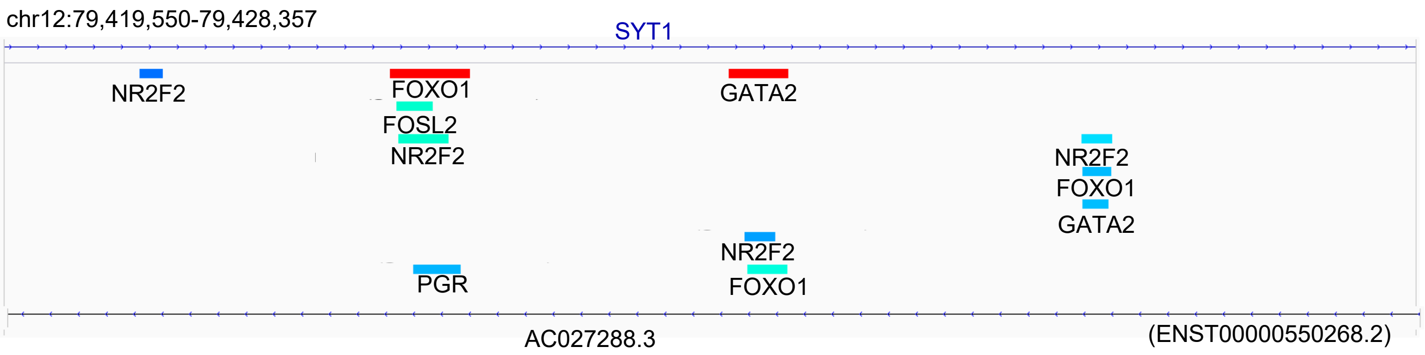

Figure S10. Transcription factor binding site cluster 2 in the first intron of ENST00000550268.2 (AC027288.3) from multiple ChIP-seq publications. A Bed file containing the data were downloaded from Chip Atlas and displayed on the IGV genome browser. A Color gradient represents $-10*\text{Log}_{10}$(MACS2 Q-value) with blue (50), cyan (250), green (500), yellow (750), and red (>1000).
